# Change in prostate volume during extreme hypo-fractionation analysed with MRI

**DOI:** 10.1186/1748-717X-9-22

**Published:** 2014-01-13

**Authors:** Adalsteinn Gunnlaugsson, Elisabeth Kjellén, Oskar Hagberg, Camilla Thellenberg-Karlsson, Anders Widmark, Per Nilsson

**Affiliations:** 1 Department of Oncology, Skåne University Hospital, Lund University, 22185 Lund, Sweden; 2 Department of Epidemiology, Skåne University Hospital, Lund University, 22185 Lund, Sweden; 3 Department of Oncology and Department of Radiation Sciences, Umeå University Hospital, SE-901 85 Umeå, Sweden; 4 Department of Oncology and Radiation Physics, Skåne University Hospital, Lund University, 22185 Lund, Sweden

**Keywords:** Hypo-fractionation, MRI, Prostate cancer, Radiotherapy, Swelling, Volume change

## Abstract

**Background:**

Hypo-fractionated external beam radiotherapy with narrow CTV-PTV margins is increasingly applied for prostate cancer. This demands a precise target definition and knowledge on target location and extension during treatment. It is unclear how increase in fraction size affects changes in prostate volume during treatment. Our aim was to study prostate volume changes during extreme hypo-fractionation (7 × 6.1 Gy) by using sequential MRIs.

**Methods:**

Twenty patients treated with extreme hypo-fractionation were recruited from an on-going prospective randomized phase III trial. An MRI scan was done before start of treatment, at mid treatment and at the end of radiotherapy. The prostate was delineated at each MRI and the volume and maximum extension in left-right, anterior-posterior and cranial-caudal directions were measured.

**Results:**

There was a significant increase in mean prostate volume (14%) at mid treatment as compared to baseline. The prostate volume remained enlarged (9%) at the end of radiotherapy. Prostate swelling was most pronounced in the anterior-posterior and cranial-caudal directions.

**Conclusions:**

Extreme hypo-fractionation induced a significant prostate swelling during treatment that was still present at the time of last treatment fraction. Our results indicate that prostate swelling is an important factor to take into account when applying treatment margins during short extreme hypo-fractionation, and that tight margins should be applied with caution.

## Background

The field of radiotherapy (RT) is rapidly evolving with new advanced treatment techniques and improved imaging. Implementation of magnetic resonance imaging (MRI) for segmentation together with sophisticated image guided radiotherapy (IGRT) techniques based on implanted fiducials has resulted in improved accuracy and precision in RT for prostate cancer [1-3]. A workflow based solely on MR, i.e. from prostate delineation to treatment planning and delivery, has been proposed and shown to reduce systematic uncertainties considerably compared to a conventional CT/MR-based workflow [4]. Evidence from prostate cancer radiotherapy trials shows that dose-escalation improves outcome [5-8] with limited increase in toxicity [9,10]. The latter is partly due to a reduction of the CTV-PTV margins compared with those applied when positioning the treatment beams based on skin marks or on bony structures [11]. In addition, inter-fraction and intra-fraction prostate motion have been studied extensively during recent years [12-14]. However, the optimal CTV-PTV margin in a specific setting is still debated [15]. When the margin is reduced to as small as 3 mm, adequate coverage of at least larger prostates seems to be jeopardized [16,17].

The CTV-PTV margin should not only take setup variations and tumour motion into account but also include any changes in the shape and size of the CTV [18]. Changes in prostate morphology during radiotherapy are not well studied. There is some evidence that prostate size increases slightly during the first week(s) after start of conventionally fractionated RT and then decreases substantially during treatment and shrinks to below baseline by the end of treatment [19,20].

Hypo-fractionated RT of prostate cancer has earned increased attention due to a proposed low α/β value, close to 1.5 Gy [21,22]. The application of higher fraction doses might result in a larger change in prostate shape and size as compared with conventional fractionation, since prostate swelling is known to occur during brachytherapy [23,24].

The aim of the present study was to measure any changes in prostate size during a course of extreme hypo- fractionation delivered with external beam technique by using sequential MRI scanning before, during and at the end of the RT course. A cohort of patients from a Swedish multicentre trial (HYPO-RT-PC), studying extreme hypo-fractionation, was used for the study.

## Methods

### Patients

Twenty patients treated with extreme hypo-fractionation were included in the present study. All patients were recruited from an on-going Scandinavian prospective randomized phase III trial (HYPO-RT-PC), which compares extreme hypo-fractionation with conventional fractionation in intermediate risk prostate cancer patients [25]. This study was approved by the local ethics committee (Division of Oncology, Department of Clinical Sciences, Lund University) and is performed according to the Helsinki Declaration of 1975, (revised in 2000). Inclusion criteria are: age < 75 years, WHO performance status 0–2, intermediate risk prostate cancer with clinical category T1c-T3a with one of the following risk factors: 1) T3a, 2) Gleason ≥ 7 or 3) PSA > 10 μg/L. PSA shall be < 20 μg/L and a biopsy-proven adenocarcinoma without any signs of spread distally or to lymph nodes are also required. Any earlier treatment for prostate cancer, previous hormonal therapy, other serious diseases (including prior malignant disease), conditions that could prevent implantation of markers into the prostate or signs of metastatic disease are exclusion criteria. Patient characteristics for the cohort in the present study are given in Table 1.

**Table 1:** Patient baseline characteristics (n = 20)

**Age**	
Median (range)	68 (59–73)
**Tumour stage**	
T1c	17
T2	3
**Gleason score**	
6	3
7	14
8	3
**iPSA (ng/mL)**	
Mean (SD)	10.2 (4.5)
**Prostate volume (cm**^**3**^)*	
Mean (SD)	73 (30)

*As segmented on CT.

### Treatment

In the HYPO-RT-PC study, patients are randomized between either conventional fractionation (39 × 2.0 Gy = 78.0 Gy given once a day, five days per week) or to an experimental arm with an extreme hypo-fractionated regimen (7 × 6.1 Gy = 42.7 Gy given every other weekday, and always including two weekends without RT). The trial arms are equieffective assuming α/β = 3 Gy, neglecting any influence of the difference in total treatment time. Both 3D-conform radiotherapy (3D-CRT) and IMRT/VMAT techniques are allowed. Hormonal treatment is not permitted.

### Radiotherapy procedure according to the HYPO-RT-PC study protocol

Three gold markers were implanted into the prostate for daily image guidance at least three weeks before the treatment planning CT to avoid post-implant oedema of the gland. Target and OAR definitions were according to ICRU [18,26,27]. The CTV, i.e. prostate (no seminal vesicles), was segmented as visualised on the treatment-planning CT (slice thickness ≤ 3 mm). CT defined prostate segmentation is mandatory according to the study protocol but MRI is recommended as an aid for target delineation. The PTV includes CTV with a 7 mm isotropic 3D-margin. The CT-based CTV volume for the patients included in the present study was already defined within the clinical trial by three different senior radiation oncologists.

### Sequential MRI scanning for CTV delineation

The patients were imaged with a Siemens Espree 1.5 T MR scanner (Siemens Medical, Erlangen, Germany) using a body coil and a T2 weighted high-resolution 3D sequence with axial slices (slice thickness 1.7-3.3 mm). This MRI sequence is used in clinical routine as aid for the CT-based target definition. The patients were placed in supine position with a leg fixation device on a flat table-top insert during the MR imaging, i.e. in the same position as for RT.

MRI scans were performed at baseline (MRI_baseline_) when the patient came for treatment-planning CT, in the middle (MRI_mid_, EQD2_3_ = 33 Gy) and at the end of treatment (MRI_end,_ EQD2_3_ = 67 Gy). The MRI studies were transferred to the treatment planning system (Nucletron Oncentra, ver 4.0) where the prostate was delineated in each MRI slice by the same radiation oncologist (AG). This delineation was done in a blinded fashion. The volume, as calculated by the treatment planning system, was registered for each CTV_MRI_. In addition, the maximum extension of the delineated prostate on the MRIs was measured in the three principal directions, i.e. left-right (x_max_), anterior-posterior (y_max_) and cranial-caudal (z_max_) to estimate any changes in size in the three directions. The x_max_, y_max_ and z_max_ values are hence the sides of the smallest rectangular prism which precisely contains the segmented prostate.

To test whether the average change in prostate volume at the various time points was significant, a standard two-sided t-test was used. A p-value < 0.05 was considered significant.

## Results

**Figure 1:**
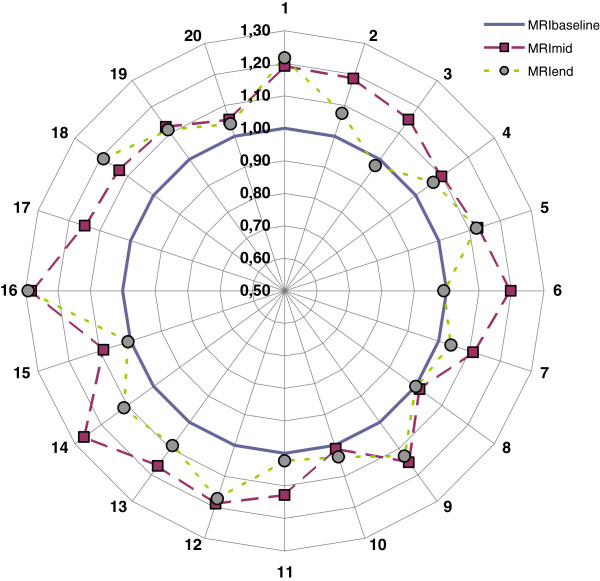
**Relative prostate volume compared to baseline (MRI**_**baseline**_**) at MRI**_**mid**_**(EQD2**_**3**_ **= 33 Gy) (squares) and at MRI**_**end**_**(EQD2**_**3**_ **= 67 Gy) (circles) for patients 1–20.**

Segmented absolute prostate volumes together with relative prostate volume changes vs. the baseline MRI volume are given in Table 2. The results are also presented graphically in Figure 1. The prostate volumes measured on the treatment-planning CT averaged 23% larger than those delineated on the baseline MRI (MRI_baseline_). The difference was statistically significant, p = 0.0001.

**Table 2:** **Prostate volumes in descending order as segmented on the treatment planning CT and on the MR images before radiotherapy (MRI**_**baseline**_**), in the middle of the treatment (MRI**_**mid**_**) and at the end of treatment (MRI**_**end**_**)**

**Pat #**	**CT**		**MRI**_**baseline**_	**MRI**_**mid**_		**MRI**_**end**_	
	**Abs. vol. (cm**^**3**^**)**	**Rel. vol.**	**Abs. vol. (cm**^**3**^**)**	**Abs. vol. (cm**^**3**^**)**	**Rel. vol.**	**Abs. vol. (cm**^**3**^**)**	**Rel. vol.**
1	35.3	1.579	22.4	26.7	1.191	27.2	1.217
2	44.5	1.369	32.5	38.6	1.187	34.9	1.074
3	33.8	0.999	33.9	39.0	1.151	33.1	0.976
4	47.8	1.105	43.3	47.5	1.098	46.2	1.067
5	45.8	1.054	43.4	48.9	1.126	48.7	1.122
6	64.8	1.455	44.5	53.3	1.198	44.1	0.991
7	71.6	1.597	44.8	49.8	1.112	46.6	1.040
8	43.5	0.906	48.0	48.7	1.015	48.0	1.000
9	79.4	1.648	48.2	55.5	1.152	54.4	1.129
10	73.0	1.511	48.4	48.9	1.011	50.1	1.037
11	59.4	1.102	53.9	60.8	1.128	55.2	1.023
12	57.0	1.037	54.9	65.3	1.189	64.4	1.172
13	--^*^	--^*^	57.2	66.6	1.166	62.2	1.089
14	83.8	1.196	70.1	88.7	1.265	78.0	1.112
15	99.0	1.347	73.5	80.0	1.088	74.1	1.008
16	79.4	1.066	74.5	95.5	1.282	96.2	1.291
17	96.5	1.145	84.3	96.8	1.148	--^‡^	--^‡^
18	105.8	1.242	85.2	96.3	1.131	101.4	1.190
19	106.2	1.021	104.0	116.8	1.123	115.6	1.112
20	153.7	1.045	147.1	155.0	1.054	152.9	1.040
**Mean**	**72.7**	**1.233**	**60.7**	**68.9**	**1.141**	**64.9**	**1.089**
SD	30.4	0.232	28.7	31.3	0.070	31.9	0.084
**p-value**^**†**^	**0.0001**	**0.0004**	**---**	**<0.0001**	**<0.0001**	**0.0008**	**0.0002**

Relative volumes and p-values are in relation to MRI_baseline_.

*Prostate was not segmented on CT as the patient had a hip prosthesis.

^‡^Missing data, MRI not performed.

^†^Paired t-test.

The median time (range) elapsed from MRI_baseline_ to MRI_mid_ and from MRI_baseline_ to MRI_end_ was 8 (6–9) days and 16 (15–17) days, respectively. According to the sequential MRI scanning analyses, extreme hypo-fractionation caused a 14% mean relative volume increase (p < 0.0001) at MRI_mid_. The mean volume increase was still present at the time of the last treatment fraction (9% at MRI_end_, p = 0.0002). There was no significant difference in mean relative volume change between prostates above vs. below the median CTV size, neither at MRI_mid_ (p = 0.30) nor at MRI_end_ (p = 0.20).

The maximum prostate dimensions (x_max_, y_max_ and z_max_) as defined above were unchanged in the lateral direction but increased in the anterior-posterior and cranial-caudal directions by 2–3 mm for MRI_mid_ or MRI_end_ as compared with baseline (see Table 3 for details). Corresponding data for “small” versus “large” prostate baseline volumes are presented in Table 4.

**Table 3:** **Average change in maximum prostate extension in lateral (∆x**_**max**_**), anterior–posterior (∆y**_**max**_**) and cranial–caudal (∆z**_**max**_**) direction (mean values and 95% CI)**

	**∆x**_**max**_**(mm)**	**P**	**∆y**_**max**_**(mm)**	**p**	**∆z**_**max**_**(mm)**	**p**
MR_mid_–MR_baseline_	0.2 (−1.1–1.5)	0.72	3.3 (1.8–4.8)	0.0002	2.5 (1.0–3.9)	0.0019
MR_end_–MR_baseline_	0.3 (−0.9–1.4)	0.60	2.0 (0.5–3.4)	0.010	2.0 (0.8–3.1)	0.0029
MR_end_–MR_mid_	0.1 (−0.8–0.9)	0.89	−1.4 (−2.7–−0.1)	0.036	−0.6 (−1.7–0.6)	0.32

**Table 4:** **Average change in maximum prostate extension in lateral (∆x**_**max**_**), anterior–posterior (∆y**_**max**_**) and cranial–caudal (∆z**_**max**_**) direction for “small”/“large” prostate volumes, i.e. below/above median MRI**_**baseline**_**volume (=50 cm**^**3**^**)**

	**∆x**_**max**_**(mm)**	**p**	**∆y**_**max**_**(mm)**	**p**	**∆z**_**max**_**(mm)**	**p**
MR_mid_–MR_baseline_	−0.5/1.0	0.24	3.3/3.3	0.98	1.9/3.0	0.44
MR_end_–MR_baseline_	−0.1/0.7	0.44	1.3/2.8	0.29	1.9/2.0	0.88
MR_end_–MR_mid_	0.0/0.0	0.41	−0.2/−0.1	0.24	0.0/−0.1	0.34

## Discussion

Variations in prostate size during a course of radiotherapy using conventional fractionation have been studied previously. Generally these studies have shown an overall prostate volume reduction at end of treatment (without any anti-hormonal treatment) as compared to baseline although with an initial volume increase [19,20,28]. Based on the relative position of implanted electro-magnetic transponders, King *et al*. showed that the prostate size increases transiently (mean 6.1%) during the first week(s) after start of conventional RT (total dose 81 Gy, 1.8 Gy/fraction) and then shrinks to below baseline by the end of treatment. The decrease in mean prostate volume was 10.9% from the first to the final day of RT. Using MRI, Nichol *et al*. studied changes in prostate size during conventionally fractionated RT (total dose 79.8 Gy, 1.9 Gy/fraction) in 25 patients. They reported a prostate volume decrease by 0.5%/fraction. Based on CT scanning at start and at the last week of RT (total dose 76 Gy, 2.0 Gy/fraction), Sanguineti *et al*. reported a mean decrease in prostate volume of 7% in 14 patients without any anti-hormonal treatment.

To our knowledge there are no earlier studies on how extreme hypo-fractionation affects the prostate volume during radiotherapy. The extreme hypo-fractionation regimen used in our study lead to a significant increase in prostate volume after three treatment fractions (EQD2_3_ = 33 Gy). This increase was still apparent at the end of treatment after six fractions (EQD2_3_ = 67 Gy). Our observations indicate that the enlargement of the CTV is both larger than that known for conventional therapy and stays enlarged during the whole treatment course. This could be an important factor to take into account when choosing margin size.

When using daily imaging for set up correction, a minimum margin size between 1.5-3 mm to compensate for intra-fraction motion of the prostate has been proposed as adequate [15,16]. Our results indicate that a margin extension of similar magnitude (covering the 95% CIs in Table 3) could be needed to take prostate swelling into account during extreme hypo-fractionation. The analysis of prostate distension showed that the prostate seemed to swell most profoundly in the anterior-posterior and cranial-caudal directions. This might indicate that a margin reduction towards the rectum should be applied with caution, especially during extreme hypo-fractionation. The difference in prostate expansion in cranial-caudal and anterior-posterior directions on one hand and lateral direction on the other hand could be due to the pelvic side wall acting as an anatomic barrier [19].

Prostate swelling during brachytherapy is well known [23,24], and thus one could expect larger swelling when using hypo-fractionation than during conventional radiotherapy treatment. Our study supports this and sparks concerns that larger treatment margins are indicated with this kind of regimen as compared with conventional treatment, especially if prostate segmentation is based on MRI only. MRI-based contouring at baseline resulted in a CTV volume that was about 20% smaller than the volume generated in the original treatment-planning CT which is in concordance with an earlier study by Smith *et al.*[29] who found an average difference of 16%. Inferior soft tissue contrast on CT as compared to MRI increases inter-observer variability in CT-based target definition which can partly explain this difference in volume between CT and MRI. The fact that current clinical evidence in prostate cancer radiotherapy is generated from CT-based target definition, implies that great care has to be taken to compensate for prostate swelling if the segmentation and treatment planning process is performed with MR-only [30]. We also looked at whether patients with larger prostate glands experienced more swelling than patients with smaller glands. No such difference in relative prostate volume change was observed.

To minimize multi-observer variation in prostate segmentation as well as MRI-sequence based errors [31], the same radiation oncologist did the delineation in a blinded fashion on the same MRI-sequence at each time-point. The fact that the prostate increased in volume at mid-treatment as compared to baseline for all patients supports that this is due to a true treatment induced swelling and not a methodological error. One could also argue that image guided set-up correction would cope with this change in prostate shape during the course of treatment. However, this correction usually involves three markers implanted centrally in the prostate gland, and thus it is probably adequate for prostate motion but less adequate for taking changes in the outer boundaries of the gland into consideration. Re-contouring of the prostate volume followed by re-planning before each fraction could be needed when using narrow margins (≤ 3 mm).

## Conclusions

Our study indicates that the prostate swells significantly during external radiotherapy when using extreme hypo-fractionation. This seems to be an important factor when defining margin size for extreme hypo-fractionation schedules for prostate cancer to minimize the risk of treatment failure when using narrow margins. In order to take prostate swelling into account when using extreme hypo-fractionation, we conclude that up to 2 mm extra margin could be needed if prostate segmentation is based only on MRI. Adaptive radiotherapy with re-planning before each fraction, which would also take changes in prostate shape into consideration, would be optimal.

We are planning a larger study on prostate volume change within the frame of the HYPO-RT-PC trial also including conventional fractionation for comparison.

### Consent

Written informed consent was obtained from all patients included in this study.

## Competing interests

All authors declare that they have no competing interests.

## Authors’ contributions

AG, EK and PN designed the study, retrieved and analysed the data and drafted the manuscript. OH gave statistical advice and revised the manuscript. CTK and AW were involved in the study design and revised the manuscript. All authors read and approved the final manuscript.
